# Nutritional supplements and herbal medicines for women with polycystic ovary syndrome; a systematic review and meta-analysis

**DOI:** 10.1186/s12906-017-2011-x

**Published:** 2017-11-25

**Authors:** Susan Arentz, Caroline A. Smith, Jason Abbott, Alan Bensoussan

**Affiliations:** 1 National Institute of Complementary Medicine, Western Sydney University, Locked Bag 1797, Penrith, NSW 2751 Australia; 2 School of Women’s and Children’s Health, University of New South Wales, Sydney, Australia

**Keywords:** Polycystic ovary syndrome, PCOS, Supplements, Vitamins, Herbal medicine, Complementary medicine

## Abstract

**Background:**

Polycystic ovary syndrome (PCOS) is a common, reproductive endocrinopathy associated with serious short and long term health risks. Many women with PCOS use ingestible complementary medicines. This systematic review examined the effect on menstrual regulation and adverse effects from randomised controlled trials.

**Methods:**

Randomised controlled trials (RCTs) that compared herbal or nutritional supplements to placebo or active controls in women with PCOS were eligible for inclusion. Electronic databases were searched to July 2017. Study selection and assessment of quality were conducted independently by two review authors.

**Results:**

Twenty four studies (1406 women) investigating seven nutritional supplements and four herbal medicines were included. No one study was assessed as having a low risk of bias. Four trials reported on the primary endpoint menstrual regulation. There was no evidence on improved menstrual regularity for calcium plus vitamin D compared to Metformin (RR: 0.66, 95% CI 0.35 to 1.23, *p* = 0.19), reduced amenorrhoea for *Camellia sinensis* compared to placebo (RR: 0.17, 95% CI 0.02 to 1.72, *p* = 0.13) and no difference in the number of menses per month for *Cinnamomum sp.* against placebo (MD 0.05, 95% CI -0.36 to 1.36, *p* = 0.26).

Adverse effects were investigated in seven studies (164 women). Mild adverse effects were found for *Cinnamomum sp.* compared to placebo (17 women, RR: 0.36, 95% CI 0.03 to 0.70, *p* = 0.03). No difference was found for adverse effects between inositol, B complex vitamins, vitamin D, chromium and placebo. Improved reproduction, metabolic hormones and hyperandrogenism was found for inositol and improved cholesterol for omega three fish oils.

**Conclusion:**

There is no high quality evidence to support the effectiveness of nutritional supplements and herbal medicine for women with PCOS and evidence of safety is lacking. High quality trials of nutritional supplements and herbal medicines examining menstrual regulation and adverse effects in women with PCOS are needed.

**Electronic supplementary material:**

The online version of this article (10.1186/s12906-017-2011-x) contains supplementary material, which is available to authorized users.

## Background

Polycystic ovary syndrome (PCOS) is the most common female reproductive endocrine disorder [1] estimated to affect up to one in five women [2]. PCOS has broad health implications including adverse metabolic (obesity, type two diabetes, cardio vascular disease), reproductive (infertility, miscarriage, pregnancy and neonatal complications) [3, 4], and psychological (anxiety, depression and stress) [5, 6] risks. The pathogenesis is underpinned by insulin resistance [7] which affects up to 75% of lean women and up to 95% of obese women (compared to 62% in BMI matched non-POCS controls) [8]. Prevention and reduction of being overweight with lifestyle interventions is first-line evidence-based treatment [9], however the strength of evidence for lifestyle is limited by high attrition in randomised controlled trials (RCTs) [10, 11] and, as in the general population, engagement and adherence to lifestyle intervention is impacted by psychosocial and physical barriers in women with established obesity [10]. Additional treatments for PCOS symptoms include pharmaceutical agents [9] (contraceptive pills (OCP) and hypoglycaemics), but contraindications for the OCP are common in overweight women [12] and hypoglycaemic agents are associated with significant rates of unpleasant side effects [13, 14], potentially worsening women’s quality of life. Women with PCOS have been shown to seek out alternative [15] and adjunct treatments including complementary medicines (CM) to improve their health, fertility and wellbeing [16].

There is some evidence of positive effects in women with PCOS for ingestible CMs including nutritional supplements [17, 18] and herbal medicines [19, 20]. Women with PCOS have significantly higher levels of homocysteine and oxidative stress [21] and nutritional supplements have demonstrated efficacy in women with PCOS [18] and risk reduction for the same conditions in other populations [22]. Endocrine mechanisms of herbal medicine may improve hormone balance in PCOS and may positively effect menstural regularity [23, 24], a critial outcome of concern for women with PCOS [25]. This study aimed to synthesise the evidence of effectiveness and safety for herbal and nutritional supplements in women with PCOS.

## Methods

RCTs that compared herbal medicines or nutritional supplements to placebo, active controls (nutritional supplements) or usual care (pharmaceutical treatment, lifestyle management) were sought. Cross-over trials were included for completeness but data from the first phase only were included in meta-analyses.

Participants were women aged between 18 and 44 with a diagnosis of PCOS according to the Rotterdam Criteria (2003), [26] or the National Institute for Health (NIH) [27].

Nutritional or herbal supplements were defined as herbal extracts in a single preparation and/or tablets containing vitamins, minerals or dietary derived molecules designed to improve symptoms associated with PCOS and not medically prescribed for conditions of deficiency [28].

The primary outcome measures were; ‘menstrual regularity’ defined as number of women with menstrual cycles of 22 to 35 days duration, number of women with amenorrhoea and number of menses per month, and ‘adverse effects’. Secondary outcomes were; secondary menstrual characteristics (ovulation, presence of a dominant follicle, ratio of luteal weeks, gonadotropin hormones), polycystic ovaries, pregnancy rates and outcomes (live births and miscarriage), hyperandrogenism (clinical and biochemical), anthropometric factors, metabolic biochemistry, PCOS associated risk factors, quality of life and anxiety and depression.

All published and unpublished RCTs comparing herbal medicine or nutritional supplements with active controls, usual care or placebo, in English language, were searched. Electronic databases included Cochrane central register of controlled trials (CENTRAL), the Cochrane Library, MEDLINE ovidSP, CINAHL, SciVerse, EMBASE, PubMed were searched from the date of inception to July 2017. Reference lists of review articles and texts were searched by hand.

Keywords were ‘polycystic ovary syndrome’, ‘PCOS’, ‘ovarian cysts’, ‘hyperandrogenism’, ‘hirsutism’, ‘acanthosis nigrans’, ‘acne’, ‘oligomenorrhoea’, ‘amenorrhoea’, ‘oligo-ovulation’, ‘anovulation’, ‘menstrual disorders’, ‘menstrual’, ‘ovulation’, ‘fertility’, AND ‘alternative’, ‘integrative medicine’, ‘complementary medicine’, ‘naturopathy’, ‘natural’, ‘herbal medicine’, ‘botanical medicine’ ‘phytomedicine’, ‘phytotherapy’, ‘herbs’, ‘diet’, ‘food’, ‘nutritional’, ‘nutrient’, ‘micro’, ‘dietary’, ‘vitamin’, ‘mineral’, ‘supplements’, ‘hyper-vitamin’, AND ‘randomis(z)ed. control trial’, ‘clinical trial’ and ‘RCT’.

Study characteristics, risk of bias and outcome data were manually extracted independently by two (of three) authors (CAS, JAA and SA) and entered onto data extraction forms adapted from the Cochrane Collaboration [29] for systematic critical appraisal. Differences of opinion were resolved by consensus. Risk of bias was assessed as high, low or unclear risk for randomization (generation of randomized schedule and allocation sequence), performance (blinding of participants), detection (blinding of outcome assessors and data analysts), attrition (reporting missing data), reporting (pre-specified and or relevant outcomes) and other areas of potential bias including factors unique to particular settings (such as study being stopped early, apparent fraudulent reporting (including the absence of declaration of funding and conflicts of interest in relation to authors affiliations) or extreme baseline imbalance) [29].

Analyses were conducted using Review Manager (RevMan) 5.3 [30]. Dichotomous data were expressed as relative risks (RR) and normally distributed continuous data as mean differences (MD). Corresponding 95% confidence intervals (CIs) and *p* values were calculated. Heterogeneity between studies was investigated by the I^2^ statistic (I^2^ of more than 50% was considered indicative of heterogeneity), the *P* value from the χ^2^ test and by visual inspection of the forest plots. For non-significant heterogeneity, results were pooled in a fixed effect model and the effects were adjusted using random effects where there was significant heterogeneity. If distributions were skewed and results reported as the median and range, with non-parametric tests of significance, the results were excluded from the meta- analysis. Androgens, cholesterol and triglycerides were converted to common measurements for comparison and the mean difference (MD) was calculated.

## Results

**Fig. 1:**
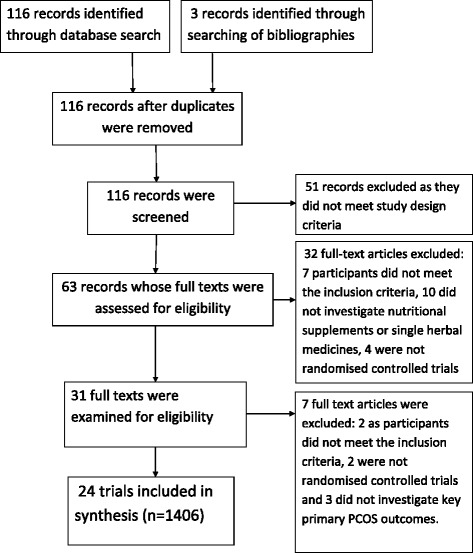
PRISMA (flow chart) diagram

A total of 116 records were identified through the electronic and manual searches. Following initial screening, 63 articles were retrieved for detailed evaluation (Fig. 1). Thirty two full text articles were excluded as they did not meet the inclusion criteria (detailed in Additional file 1: Table S1). Following detailed evaluation of 31 full text studies, a further seven studies were excluded due to not investigating key signs and symptoms of PCOS, not being randomised or not including participants with a clinical diagnosis (detailed in Additional file 1: Table S1). Twenty four randomised controlled trials met the inclusion criteria [31–54] including 1406 women.

### Included studies

#### Design

The sample sizes ranged from 10 [43] to 283 [37]. All studies were conducted at university hospitals or clinical research centres in America [42, 43, 46, 51, 52], Australia [35], Egypt [49], Hong Kong [33], Iran [32, 40, 44, 47, 48, 50, 54], Italy [31, 34, 36–38], Turkey [53], United Kingdom [39] and Venezuela [41, 45].

#### Participants

Five studies included overweight participants (BMI greater than 25) [33, 35, 44, 45, 51], and two studies included women of normal bodyweight [36, 41]. One study stratified randomization according to BMI (overweight or normal weight) [47]. Seventeen studies excluded participants with other endocrine disease [42, 46, 53, 54] and elevated serum prolactin (PRL) [31–33, 35, 37, 38, 41, 44, 46–48, 50–52].

#### Interventions

Seven nutritional supplements and four herbal medicines were investigated (Table 1).The treatment durations ranged from thirty days [39] to seven months [49] with most treatment durations (13 studies) of eight to twelve weeks (Table 1). Inositol was investigated in eight studies and three investigated omega three fish oils. Calcium plus vitamin D, vitamin D alone, selenium and *Cinnamomum sp.* were investigated in two studies and nutritional supplements B complex vitamins, chromium, and herbal medicines *Camellia sinensis*, *Mentha spicata* and *Cimicifuga racemosa* were investigated in single studies. Calcium plus vitamin D, B complex, inositol, selenium and *Cimicifuga racemosa* were compared with pharmaceutical controls (metformin and clomiphene citrate) [47–50, 53, 54].

**Table 1:** Interventions: Nutritional supplements and herbal medicine

	Reference	Dose	Control	Duration
Omega 3 fish oils	Cussons A J; Watts GF; Mori TA; Stuckey BGA. 2009 [34]	4000 mg per day EPA 675 mg; DHA 1400 mg (Ocean nutrition® by Halifax, Nova Scotia, Canada)	Placebo Olive oil 4000 mg (Cardinal Health Australia, Vic Australia)	8 weeks
	Mohammadi E; Rafraf M; Farzadi L; Asghari-Jafarabadi M; Sabour S. 2012 [43]	4000 mg omega 3 (EPA 720 mg; DHA 480 mg) (Good Health Company, USA)	Placebo liquid paraffin 2000 mg in four capsules (Zihravi Pharma, Iran)	8 weeks
	Vargas LM; Almario RU; Buchan W; Kim, K; Karakas SE. 2011 [50]	3500 mg (EPA: 2450 mg; DHA: 1210 mg) (Nordic Naturals Watsonville, California)	Placebo (soybean oil 3500 mg) (Nordic Naturals, Watsonville California)	6 weeks
Chromium	Lucidi SR; Thyer AC; Easton CA; Holden AEC; Schenken RS; Brzyski RG. 2009 [42]	Chromium picolinate 200mcg per day (manufacturer not reported)	Placebo (type or manufacturer was not reported)	16 weeks
Selenium	Hosseinzadeh F; Hosseinzadeh-Attar M; Yekaninejad J; Saeed M; Rashidi B. 2016 [39]	Selenium 200mcg per day (Nature Made Pharmaceutical Company, California USA)	Placebo (type not specified) (Roshd Pharmaceutical Incubation Centre, Tehran University, Iran)	12 weeks
	Razavi M; Jamilian M; Kashan Z; Fakhrieh HZ; Mohseni M; Ghandi Y; Bagherian T; Asemi Z 2015 [47]	Selenium 200mcg plus Metformin 1500 mg per day (Nature Made Pharmaceutical Company, California USA)	Placebo (cellulose) plus Metformin 1500 mg per day(Barij Essence Pharmaceutical Company, Iran)	8 weeks
Vitamin D	Ardibilli HR; Gargari BG; Farzadi L. 2012 [31]	Vitamin D. Three oral treatments of 50,000 IU per 20 days (D-vitin® by Zahravi Pharma Company, Iran)	Placebo, One tablet every 20 days (Zihravi Pharma Company, Iran)	2 months
	Raja-Khan N; Shah J; Stetter CM; Lott MEJ; Kunselman AR; Dodson WC; Legro RS. [45]	Vitamin D 12000 IU per day(Cholecalciferol in soy lecithin) (Maximum D3® by BTR Group, USA)	Placebo per day (soy lecithin without cholecalciferol) (BTR Group, USA)	12 weeks
Vitamin D plus Calcium	Rashidi R; Haghollahi F; Shariat M; Zayerii F. 2009 [46]	Calcium 1000 mg + vitamin D 400mcg per day (Cal D tablets by Tehran Derou, Iran)	Metformin 500 mg, three times per day (Minoo Darou, Iran)	12 weeks
	Tehrani, HG; Mostajeran F; Shahsavari S. 2014 [49]	Calcium 1000 mg (Osvah Pharmaceuticals) Vitamin D 50,000 IU per fortnight (Zahravi Pharmaceutical company)	Metformin (Sobhan darou pharmaceutical company)	16 weeks
Vitamin B complex	Kilicdag EB; Bagis T; Tarim E; Aslan E; Erkanli S; Simsek E; Haydardedeoglu B; and Kuscu E. 2005 [52]	B1: 500 mg; B6: 500 mg; B12: 2000mcg + Metformin 1700 mg/day, (Apikobal® by Santa Farma Turkey) (Glugophage® by Merke, Turkey)	Metformin 1700 mg/day (Glucophage® by Merke Turkey)	12 weeks
Inositol (vitamin B8)	Artini PG; Di Berardino OM; Papini F; Genazzani AD; Simi G; Ruggiero M; Cela V. 2013 [30]	Inositol 2 g and folic acid 200 mg plus folic acid 200mcg (Inofert® by Ital Pharmaco, Milano, Italy)	Folic acid 400mcg daily (Inofert® by Ital Pharmaco, Milano, Italy)	12 weeks
	Costantino D: Minozzi G; Minozzi F; Guaraldi C. 2009 [33]	Inositol 4 g + folic acid 400mcg (Inofolic®)	Folic acid 400mcg (Fertifol®)	12–16 weeks
	Dona G; Sabbadin C; Fiore C; Bragadin M; Giorgino FL; Ragazzi E; Clari G; Bordin L; Armanini D. 2012 [35]	Inositol 1200 mg/day as powder, pre-dosed presented in sachets dissolved in water (manufacturer not reported)	Placebo (matched powder type or manufacturer not reported)	12 weeks
	Gerli M; Mignosa; Di Renzo GC. 2003 [36]	Inositol 200 mg (Gestosan®LO.LI. Pharma, Rome, Italy)	Placebo matched to Gestosan®. (Type or manufacturer not reported)	20 weeks
	Gerli E; Papaleo A; Ferrari GC; Di Renzo GC. 2007 [37]	Inositol 4 g plus folic acid 400mcg (Inofolic®LO.LI. Pharma, Rome, Italy)	Folic acid 400mcg (manufacturer not specified)	20 weeks
	Iuorno MJ; Jakubowicz DJ; Baillargeon JP; Dillon P; Gunn RD; Allan G; Nestler JE 2002 [40]	Inositol (chiro) 600 mg (INS-1® by Insmed pharmaceuticals, Richmond, Virginia, USA)	Placebo (type or manufacturer not reported)	6–8 weeks
	Jamilian M; Farhat P; Foroozanfard F; Ebrahimi FA; Aghadavod E; Bahmani F; Badehnoosh B; Jamilian H; Asemi Z [54]	Inositol 4 g + folic acid 400mcg (LO.LI. Rome, Italy)	Metformin (Tehran Darou Pharma. Tehran, Iran)	12 weeks
	Nestler JE; JakubowczDJ; Reamer P; Gunn R; Allan G. 1999 [44]	Inositol 1200 mg labelled according to subject number (INS-1® by Insmed pharmaceuticals, Virginia, USA)	Placebo Labelled and packaged at the same time as inositol. Type not reported (Insmed pharmaceuticals USA)	6–8 weeks
*Camellia sinensis*	Chan CW; Marcel Koo MWL; Ng EHY; Tang OS; Yeung WSB, Ho P-C. 2006 [32]	*Camellia sinensis* tea infused in boiled water for 30 mins, freeze dried and encapsulated. Epigallocatechin (EGCG) standardised to 373.92 mg ± 20.57, equivalent to 1.5 cups of tea.	Placebo capsules Identical in appearance. Type or manufacturer not specified.	12 weeks
*Cimicifuga racemosa*	Shahin AY; Mohammed SA. 2014 [48]	120 mg *Cimicifuga* extract plus clomiphene 150 mg on menstrual cycle days 3–7, trigger injection (10,000 IU, intramuscular Human Chorionic Gonadotropin) and progesterone supplementation (50mcg per day for two days following trigger injection) (Klimadynon® Bionorica, Neumarkt, Germany) (Pregnyl, Organon, Holland, The Netherlands)	Clomiphene, 150 mg per day administered on menstrual cycle days three to seven, a trigger injection (HCG) and progesterone support (50mcg per day for two days following trigger injection). (Pregnyl, Organon, Holland, The Netherlands)	Three alternate menstrual cycles
*Cinnamomum sp.*	Wang JG; Anderson RA; Graham GM; Chu MC 2007 [51]	*Cinnamomum sp.* 1000 mg per day (Integrity Nutriceuticals, Sarasota, Florida USA)	Placebo (Type or manufacturer were not reported)	8 weeks
	Kort DH; Lobo RA. 2014 [41]	*Cinnamomum sp.* 1500 mg per day (Cinnulin PF Integrity Nutraceuticals International, USA)	Placebo (identical) (Integrity Nutraceuticals International)	6 months
*Mentha spicata*	Grant, P. 2009 [38]	Tea bags standardised content of dried *Mentha spicata* leaves. Uniform instructions for preparation provided to participants	Chamomile tea uniform preparation instructions provided.	30 days

#### Outcomes

Four studies reported on the primary outcome, menstrual regularity [33, 42, 47, 50]. Seven trials reported on adverse effects (164 women) [41–43, 45, 46, 52, 53]. Seven studies reported on secondary menstrual cycle characteristics [34, 37, 38, 41, 45, 49, 50]. One trial reported on polycystic ovaries [49]. Three studies reported on pregnancy rates [47–49] and one on miscarriage [49]. Fourteen trials reported on the outcomes of biochemical hyperandrogenism [31, 33–36, 39–43, 45, 48, 51, 54]. Four trials reported on the outcomes of clinical hyperandrogenism with validated tools including the modified Ferriman Gallwey score to measure degree of hirsutism [39, 48, 50, 54], and acne with the dermatology quality of life index for health related quality of life [39]. Eight trials reported anthropometric outcomes of participants [33, 37, 38, 44, 50, 52–54]. Eighteen trials reported metabolic biochemistry including fasting glucose and insulin [31–38, 40–46, 51–53].

### Risk of bias for included studies

**Fig. 2:**
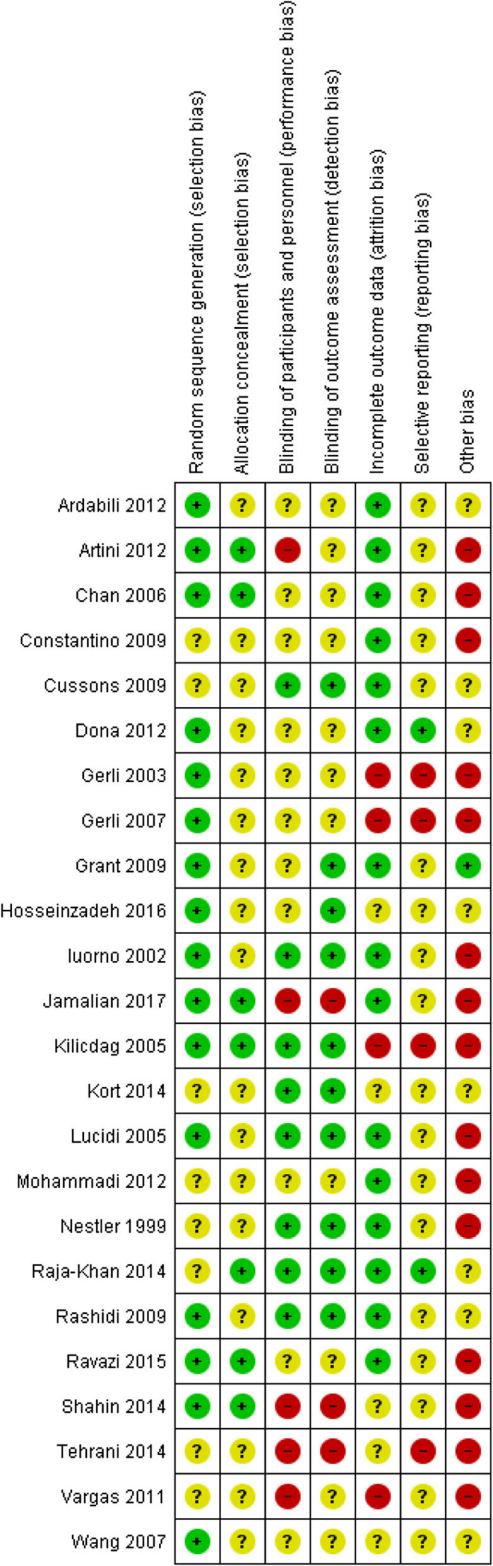
Risk of Bias summary

All 24 studies were assessed as having some risk of bias (Fig. 2). Incomplete or poor reporting by most trial authors impacted on the assessment, and consequently an unclear risk of bias for at least two characteristics was determined in 23 studies [31–52, 54]. Seven studies were found to have high risks of bias in at least three domains including performance [49–51, 54], detection [49, 50, 54], attrition [37, 38, 51, 53], reporting [37, 38, 50, 53] and other forms of bias [37, 38, 49–51, 53, 54]. Fourteen studies were at high risk for other sources of bias including nine with imbalanced baseline characteristics [31, 33, 37, 38, 40, 41, 43–45, 54], insufficient time to detect change [32, 34, 39], and an active control reported as placebo (placebo consisted of lecithin which is a natural source of inositol [55]) [17]. Three studies did not report the effect of high attrition (over 30%) on baseline menstrual characteristics in the test and control groups [37, 38, 42]. Twelve studies declared conflicts of interest [31, 32, 35, 36, 42, 44–46, 48, 49, 51, 54]. Ten studies reported no conflicts of interest and two studies reported potential conflicts of interest due to authors’ affiliations [46; 45]. Fourteen studies reported sources of study funding [32, 35, 36, 40–49, 51]. Three studies were funded with grants from the pharmaceutical industry [35, 41, 45], twelve studies were funded by university or research institute grants [32, 36, 40, 42–49, 51] and one study was funded by both pharmaceutical industry and an institute grant [45].

### Effects of interventions

Data from two trials were not included in analyses. One trial used a cross-over trial design and did not report first phase outcomes [35]; the other study reported outcomes as the median rather than the mean [33]. Where data could not be included in a meta-analysis a narrative reporting of the findings was presented.

#### Nutritional supplements for PCOS

##### Calcium plus vitamin D

Two trials (78 women) compared calcium plus vitamin D with metformin for the number of women with regular menstrual cycles (21–35 days) [47, 50]. There was no evidence of an improved effect (RR 0.66, 95% CI 0.35 to 1.23, *p* = 0.19, I^2^ 0%). Adverse effects were not reported.

*Secondary outcomes*

Rashidi and colleagues reported on the number ovarian cysts defined as immature follicles (diameter 5-9 mm) and pregnancy rates for calcium plus vitamin D against metformin [47]. No difference between groups was found. Tehrani and colleagues reported on BMI and hirsutism (defined as Ferriman Gallwey score over 8) [50]; no difference was found between groups.

##### Vitamin B complex

One study investigated B complex vitamins plus metformin compared with metformin alone, for 32 women [53]. Results were reported as ‘women taking Metformin’ and between group differences were not reported. No data were available for B complex vitamins on menstrual regularity. No difference for adverse events was found.

*Secondary outcomes*

Four secondary outcomes for vitamin B complex plus metformin were reported. No significant effects were found for pregnancy rates, waist to hip ratio or HOMA-IR.

##### Inositol (vitamin B8)

No trials reported on the primary outcome menstrual regularity for inositol (Table 2). Two trials (64 women) reported on adverse events for inositol [41, 45]. No adverse effects were found for inositol against placebo [41, 45].

**Table 2:** Inositol for reproduction, hyperandrogenism, metabolism, anthropometry and risk factors

	Treatment n^*^(number of effects)/N	Control n^*^ (number of effects)/N	Number of RCTs Heterogeneity (I^2^, *p* value)	Treatment effect MD or RR	95% CI	*p* value
Hyperandrogenism						
Free testosterone nmol/l	55	51	3 [22, 23, 30] 0%, *p* = 0.94	MD −0.02	−0.10 to 0.06	0.62
Total testosterone^**^ nmol/l	103	89	5 [34, 36, 41, 45, 54] 32%, *p* = 0.0009	MD 0.99	−1.54 to −0.44	< 0.001
Androstenidione nmol/l	73	59	4 [22, 23, 30, 37] 0%, *p* = 1.0	MD 3.59	−4.62 to −2.55	< 0.001
Sex hormone binding globulin (SHBG) nmol/l	55	67	2 [22, 23] 0%, 0.50	MD 40.67	25.83 to 55.50	< 0.001
Modified Ferriman Gallwey score	30	30	1 [54]	MD 0.90	−1.18 to 2.98	0.40
Reproduction						
Number of days to ovulation (number of days)	181	194	2 [24, 25] 0%, *p* = 0.90	MD 17.7	−31.4 to −4.1	0.01
Ratio of luteal week to total trial luteal weeks: total trial weeks	166/697	140/1000	2 [24, 25] 0%, *p* = 0.90	RR 1.7	1.39 to 2.08	< 0.001
Number of ovulations	25/32	8/32	2 [22, 23] 0%, *p* = 0.95	RR 3.13	1.68 to 5.83	0.001
Number of women who did not ovulate	16/181	34/194	2 [24, 25] 0%, *p* = 0.95	RR 0.5	0.29 to 0.86	0.01
Pregnancy	18/71	6/63	3 [24, 25, 36] 0%, *p* = 0.95	RR 2.78	1.19 to 6.5	0.02
Live births	8/25	3/25	1 [36]	RR 2.67	0.80 to 8.90	0.11
FSH mIU/l	25	25	1 [36]	MD 1.4	−1.63 to −1.17	0.001
LH mIU/l	25	25	1 [36]	MD 3.5	−4.9 to −2.1	0.001
FSH:LH ratio	25	25	1 [36]	MD 0.4	−0.68 to −0.12	0.006
Anthropometric						
BMI^***^	48	38	2 [37, 54]	MD −0.30	−1.10 to 0.50	0.46
	57	57	3 [22, 23, 36] I^2^ 31%, *p* = 0.24	MD 0.40	−0.07 to −0.86	0.86
Waist to hip ratio	32	32	2 [22, 23] 0%, *p* = 0.32	MD −0.02	−0.03 to −0.01	0.01
Metabolic hormones						
Fasting glucose mmol/l	73	59	4 [22, 23, 30, 37] 0%, *P* = 0.52	MD 1.17	−1.72 to – 0.63	< 0.001
Fasting insulin μU/ml	73	59	4 [22, 23, 30, 37] 18%, *p* = 0.30	MD 2.7	−3.9 to −1.5	< 0.001
HOMA-IR^****^	25	25	1 [36]	MD −1.30	−1.60 to −1.00	< 0.001
	18	8	1 [37]	MD −0.60	−0.89 to – 0.30	< 0.001
Risk factors						
Cholesterol mmol/l	32	32	2 [22, 23] 27%, *p* = 0.24	MD 0.56	−1.07 to −0.04	0.04
Triglycerides mg/dL	32	32	2 [22, 23] 8%, *p* = 0.30	MD 31.6	−61.1 to −2.0	0.04
HDLmg/dL	22	22	1 [22]	MD 0	−4.73 to 4.73	1.0
LDL mg/dL	22	22	1 [22]	MD −2.0	−13.7 to 9.7	0.74
CRP	30	30	1	MD −2.2	−3.57 to −0.83	< 0.01

*calculated for dichotomous outcomes only

**five trials with significant heterogeneity (I^2^ = 75%, *p* = 0.003) following analyses incorporating random effects

***five trials with significant heterogeneity (I^2^ = 89%, *p* < 0.0001) following analyses incorporating random effects, and not combined

**** two trials with significant heterogeneity (I^2^ = 91%, *p*-0.0009) following analyses incorporating random effects, and not combined

*Secondary outcomes*

**Fig. 3:**
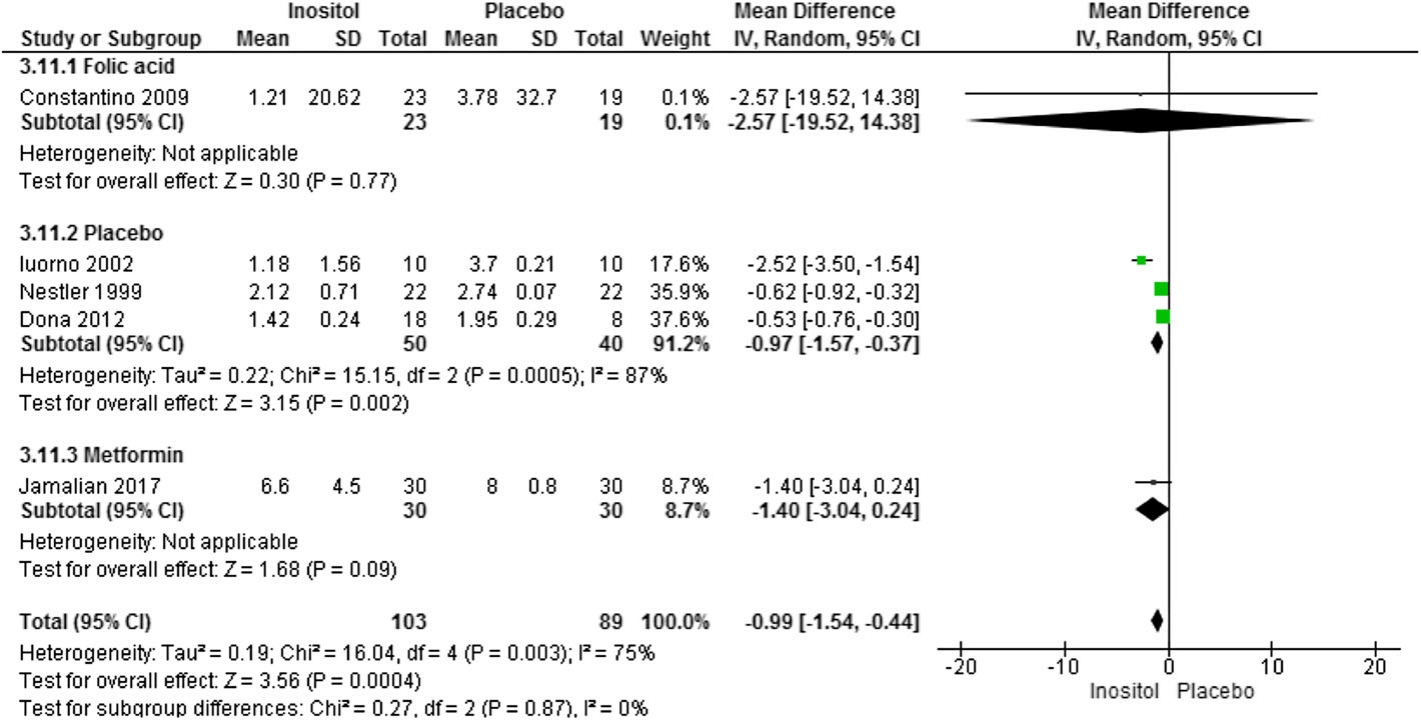
Meta-analysis inositol versus placebo for total testosterone in women with PCOS

Significant benefits were found for inositol versus placebo and active controls (90 women) for the secondary outcomes of reproductive characteristics and hyperandrogenism (Fig. 3) and (Table 2).

The number of ovulations was significantly higher for women taking inositol (two studies, 64 women, RR 3.13, 95% CI 1.68 to 5.83, *p* < 0.001). Two trials reported on a subgroup of women wanting to conceive (84 women) [37, 38] and a third trial included women preparing to undergo medically assisted fertility treatment (50 women) [31]. Meta analyses of these three studies (134 women) demonstrated pregnancy rates were significantly higher for women taking inositol compared to controls (RR 2.8, 95% CI 1.2 to 6.5, *p* = 0.02).

Significant benefits were found for the secondary outcomes of total testosterone, (five studies, 192 women, MD 0.99 nmol/l, 95% CI -1.54 to −0.44, *p* < 0.001) [34, 36, 41, 45, 54] (Fig. 3) and androstenidione (four studies, 132 women, MD 3.59 nmol/l, 95% CI -4.6 to −2.6, *p* < 0.001) [34, 36, 41, 45] and SHBG (two studies, 122 women, MD 40.67 nmol/l, 25.8 to 55.5, *p* < 0.01) [41, 45]. No significant benefit was found for free testosterone following treatment with inositol [34, 36, 41, 45] or for clinical hyperandrogenism (Ferriman Gallwey score) for inositol compared with metformin [54].

Significant benefits were found for fasting glucose (four trials, 132 participants, MD-1.17, 95% CI -1.72 to −0.63, *p* < 0.01), [34, 36, 41, 45]; fasting insulin (four trials, 132 participants, MD −2.7 μU/ml, 95% CI -3.9 to −1.5, *p* < 0.01) [34, 36, 41, 45] and reduced insulin resistance (HOMA-IR) (two trials, 76 participants, MD -1.3, 95% CI -1.6 to −1.0, *p* < 0.01) [31, 36] (Table 2). Significant heterogeneity was found for the two trials investigating inositol for HOMA-IR (I^2^ = 91%, *p* < 0.001) and effects were adjusted using a random effects model. Study heterogeneity was likely due to different characteristics of participants at baseline (normal weight [36] and overweight [31]), different doses of inositol and types of controls (1200 g per day versus placebo [36] and 2 g plus folic acid versus folic acid alone [31]).

Significant benefits were found for the secondary outcome waist to hip ratio (two studies, 64 women, MD -0.02, 95% CI -0.03 to −0.01, *p* = 0.01) [41, 45]. Mixed results were found for inositol with BMI in five trials [31, 36, 41, 45, 54] (Table 2). One trial showed significant improvements in BMI [36], whilst three others found no significant difference between groups [31, 45, 54]. A fifth trial found significantly increased BMI for women taking inositol compared to placebo controls however this study included lean participants and mean BMI at endpoint remained within normal BMI range (22.3 ± 0.3) [41]. Significant heterogeneity of trials prevented meta-analysis (I^2^ = 89%, *p* < 0.00001). The high heterogeneity may be explained clinically with variable baseline BMI categories of participants ranging from normal [36, 41, 54], overweight [31, 54] and obese [45]. Dose variation of inositol may have also contributed to study heterogeneity with daily doses of 600 mg [41], 1200 mg [36, 45] and 2 g [31] [36]) and durations of treatment for six [41, 45] and twelve weeks [31, 36, 54]) [54]. Significant benefits were found for total cholesterol (two trials, 64 women, MD 0.56 mmol/l, 95% CI -1.07 to −0.04, *p* = 0.04) [41, 45] and triglycerides (two trials, 64 women, MD 31.6 mg.dl, 95% CI -61.1 to −2.0, *p* = 0.04) [41, 45] following treatment with inositol compared to placebo controls. Reduced inflammation (c-reactive protein (CRP)) was found for inositol compared to metformin in one study (MD -2.2, 95% CI -3.57 to −0.83, *p* < 0.01) [54].

##### Chromium supplements

One small study reported on chromium compared to placebo in ten women with PCOS [43]. The study did not report on menstrual regulation. No adverse events were reported for either chromium or placebo. Five secondary outcomes free testosterone, total testosterone, ovulation, FSH and LH were reported. No significant treatment effects were found for any outcomes.

##### Vitamin D

Two RCTs examined vitamin D supplements compared with a placebo in 78 women with PCOS [32, 46]. Heterogeneity of studies was not significant (I^2^ = 0%) and data were pooled for analyses. The effects of vitamin D on menstrual regulation were not reported. No adverse effects were found in one study [46].

*Secondary outcomes*

No significant treatment effect was found for vitamin D compared with placebo for metabolic hormones including fasting glucose, fasting insulin, insulin resistance or insulin sensitivity [32, 46]. One study including 28 women found no evidence of improvements for vitamin D compared to placebo for free and total testosterone, cholesterol and high and low density lipoproteins triglycerides and inflammation (CRP) [46].

##### Omega three fish oils

Two studies investigated omega 3 supplements against placebo in 95 women [44, 51]. Neither study reported on menstrual regulation or adverse effects.

*Secondary outcomes*

**Fig. 4:**
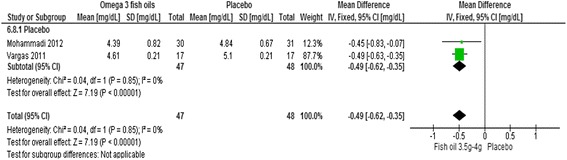
Meta-analysis omega three fish oils for cholesterol in women with PCOS

A significant effect was found for the risk marker total cholesterol in women receiving omega 3 fish oils compared to placebo controls (two trials, 95 women, −0.49 mmol, 95% CI -0.62 to −0.35, *p* < 0.001) [44, 51] (Fig. 4).

No evidence of an effect was found for free testosterone or SHBG [51], waist to hip ratio [44] or for inflammation (CRP) [44]. Findings were mixed for some secondary outcomes including triglycerides, HDL and LDL [44, 51], BMI [44, 51], fasting glucose and insulin resistance [44, 51]. Clinical heterogeneity was likely due to the dose of omega three. One trial received treatment of 3.5 g (EPA 258 mg and DHA 242 mg) for six weeks [51], which was less than the other trial where women received 4 g (EPA 720 mg and DHA 480 mg) over eight weeks and additionally due to wide variation in mean BMI at baseline. Both trials included women with a BMI that ranged between 25 and 40–45 however the mean BMI for women in one trial was in the overweight category (BMI 28) [51] compared to morbidly obese (BMI 36) in the other [44]. Significant effects were found for morbidly obese women taking the higher dose of fish oils [44] for fasting glucose (−0.33 mmol/l, 95% CI -0.26 to −0.40, *p* < 0.001), insulin resistance (HOMA-IR) (−1.23, 95% CI -1.6 to −0.86, *p* < 0.001) and reduced LDLs (−0.88, 95% CI -1.04 to −0.72, *p* < 0.01) [44].

##### Selenium

Two studies investigated selenium supplements against placebo in 109 women [40, 48]. One investigated added metformin in both study groups [48]. Neither menstrual regulation nor adverse effects were investigated in either study.

*Secondary outcomes*

A significant effect was found in one study for selenium against placebo in 53 women for fasting glucose (MD 4.66 mg/dl, 95%CI 3.23 to 6.09, *p* < 0.00001), insulin resistance (HOMA-IR) (MD 0.24 95%CI 0.06 to 0.42, *p* = 0.008), total testosterone (MD 0.18 ng/ml, 95%CI 0.09 to 0.27, *p* < 0.0001), SHBG (MD 12.80 nmol/l, 95% CI 6.22 to 19.38, *p* < 0.0001) and FAI (MD 3.93, 95% CI 3.54 to 4.32, *p* < 0.00001) [40]. No effect was found for fasting insulin. Ravazi and colleagues [48] examined selenium in addition to pharmaceutical management (metformin) against metformin plus placebo in 64 women. No evidence of an additive effect was found for free testosterone, inflammation or pregnancy rates.

##### Herbal medicines for PCOS

One trial reported on *Camellia sinensis* (green tea) capsules compared with placebo for menstrual regularity [33]. Chan and colleagues (2006) investigated the proportion of amenorrheic women over a three month period (34 women) [33]. No difference was found between groups. Adverse effects were not investigated.

*Secondary outcomes*

Chan and colleagues (2006) reported on secondary outcomes however data were presented as median differences and the interquartile range and analysed for changes over time. Consequently data were not suitable for inclusion in analyses in this review [33]. No significant differences in median values over time were found for women taking *Camellia sinensis* or placebo for testosterone, SHBG, FAI, androstenidione, FSH, LH, BMI, waist to hip ratio, insulin, glucose, cholesterol, triglycerides HDL or LDL.

##### Cimicifuga Racemosa

One study examined the combination of *Cimicifuga racemosa* and the pharmaceutical agent Clomiphene citrate (clomiphene) against clomiphene alone in 194 women [49]. Menstrual regulation or adverse effects were not reported.

*Secondary outcomes*

A significant treatment effect was found for the combination group for reduced days to ovulation (MD −3 days, 95% CI -3.51 to −2.49, *p* < 0.01), pregnancy rates (RR 1.98, 95% CI 1.19 to 3.31, *p* < 0.01), FSHmIU/l (MD 0.40, 95% CI 0.22 to 0.58, *p* < 0.01), LH mIU/l (MD -2.3, 95% CI -2.55 to −2.05, *p* < 0.01), and reduced miscarriage (RR 0.62, 95% CI 0.22 to 1.74, *p* = 0.36).

##### Cinnamomum sp.

No evidence of a menstrual regulation effect was found in one study investigating *Cinnamomum sp.* compared with placebo in 17 women [42]. Four non-serious adverse events were reported for *Cinnamomum sp.* [42]. These included headache, heartburn, menstrual cramps, nausea and diarrhoea. No adverse events were reported in the second study by Wang and colleagues [52]. Use of a variable dose may explain the different findings for adverse effects; one investigated 1500 mg of *Cinnamomum sp.* per day over six months [42] and the other 1000 mg per day over eight weeks [52].

*Secondary outcomes*

Two studies reported on metabolic hormones following treatment with *Cinnamomum sp.* compared to placebo [42, 52]. Primary data were not reported by Wang and colleagues and between groups could not be examined [52]. No evidence of a treatment effect for *Cinnamomum sp.* was found for insulin resistance (HOMA-IR) or insulin sensitivity (QUICKI) [42].

##### Mentha Spicata

No data on primary outcomes were reported.

*Secondary outcomes*

A significant treatment effect was found for *Mentha spicata* compared to *Matricaria recutita* (chamomile) tea for reduction in total testosterone in 41 women (MD 0.18 nmol/l, 95% CI -0.34 to −0.02, *p* = 0.03) [39]. No significant treatment effects were found for free testosterone, hirsutism or the dermatology index quality of life assessment.

## Discussion

This systematic review included 24 randomised controlled trials of 1406 women with PCOS. Seven nutritional supplements and four herbal medicines were investigated and meta-analyses were reported for calcium plus vitamin D, *Cinnamomum sp.*, inositol (vitamin B8), omega three fish oils and vitamin D. Menstrual regularity, a defining feature of critical interest to women with PCOS [25], was examined in only four studies and no significant treatment effect compared to controls was found. However secondary outcomes of time to ovulation, ovulation rates, hyperandrogenism, reproductive and metabolic hormones, waist to hip ratio, cholesterol and triglycerides were significantly improved by inositol and total cholesterol was significantly lowered by omega three fish oils compared to controls. Adverse effects were reported in seven studies for four nutritional supplements and one herbal medicine. Mild adverse effects were found for *Cinnamomum sp*. There were no long term investigations of safety.

### Findings for the primary outcome, menstrual regulation

This review found no single nutritional supplement or herbal medicine significantly improved the primary outcome, menstrual regulation. The oral contraceptive pill (OCP) is first line pharmaceutical treatment for menstrual regularity in women with PCOS [9], however it is contraindicated in women with increased cardiovascular risks [12, 15] and women with PCOS have reported preferences for effective alternatives [15]. Metformin is an effective alternative however women with PCOS have reported high rates of unpleasant side effects [13].

There is low quality evidence from RCTs for menstrual regulation included in this review. Trials were characterized by a high risk of selection, performance and detection biases and an unclear risk of reporting bias. Evidence from methodologically sound RCTs is needed to examine the reliability of findings for calcium plus vitamin D compared with metformin for menstrual regulation.

Chan (2006) and colleagues found a treatment effect on the primary outcome in a sub-analysis investigating *Camellia sinensis* with a lower proportion of amenorrheic women at twelve weeks for women taking *Camellia sinensis* compared to placebo controls, however differences between groups were not significant [33]. Findings were limited by the small sub-group sample size (only 14 women), the study was underpowered for the number of amenorrheic women and was assessed as low quality due to not reporting baseline characteristics of subgroups and for reporting on medians rather than means.

The findings of this review contribute to the evidence for Chinese Herbal Medicine (CHM) for oligomenorrhoea and amenorrhoea in women with PCOS [23]. Traditional Chinese herbal formulations are another form of ingestible complementary medicine of interest to women with PCOS [16]. In that systematic review which included Chinese and English language papers, only one small, low quality study investigated menstrual cyclicity outcomes for CHM and found an improved menstrual regulation response from the oral contraceptive pill (Diane 35) when combined with CHM compared to Diane 35 alone (RR 2.6, 95% CI 1.06 to 6.39, *p* = 0.04). We have some low grade evidence to add to women’s treatment decisions for menstrual regularity; calcium plus vitamin D, compared to metformin does not appear to provide any further benefits on this outcome. We agree that the evidence for ingestible CMs for menstrual regulation is poor quality and inconclusive due to the absence of findings from methodologically sound clinical studies.

### Findings for the nutritional supplement inositol

This review provides some support for a therapeutic role of inositol. As an essential structural component of cell membranes, inositol is needed for cell membrane signalling functions including those involving neurological signalling in the brain, second messenger systems and insulin signal transduction. Impaired insulin signalling in skeletal muscle and adipose tissue has a pathogenic role in conditions of insulin resistance [56, 57]. It has been proposed that ovarian theca cells of women with PCOS remain sensitive to insulin despite insulin resistance, and theca cell androgen production continues through an alternative insulin signal transduction system [58]. Evidence for a range of doses of inositol suggests therapeutic supplementation may improve metabolic profile in women with PCOS in addition to reduced biochemical hyperandrogenism and improved ovulation and pregnancy rates. However, only one small study included in this review examined live birth rates and no significant improvements were found compared to controls. As healthy, live birth outcomes are the primary goal of all interventions for women with PCOS seeking treatment for infertility there is a need for more high quality randomised controlled trials to report on this outcome. Adverse effects of inositol were examined in only two studies. Despite the likely low risks [59] this systematic review found no long term investigations assessing safety, and the evidence of safety is not established in women with PCOS.

The findings of this review differ with the systematic review of hypoglycaemic interventions for women with PCOS [14]. Tang and colleagues found no evidence for inositol compared with placebo in two studies for improved ovulation in 327 women (Odds Ratio 5.38, 95% CI 0.70 to 41.31, I^2^ 81%) [38, 45], or for lowered androgens, metabolic markers or blood lipids in 44 women [45]. Differences in our findings may be explained by the variable study inclusion criteria and the number of studies included in analyses. Tang and colleagues included studies only investigating the d-chiro isomer form of inositol against placebo or no treatment [14]. This present review included studies investigating any isometric form of inositol and active controls.

### Well-being and quality of life outcomes

Women are likely to use nutritional supplements and herbal medicines in addition to conventional pharmaceutical management possibly to improve well-being and quality of life [16, 60]. No studies investigated well-being or quality of life (QoL) outcomes and few studies examined safety for the use of nutritional and herbal supplements in conjunction with pharmaceutical treatment. Further evidence is needed to inform women’s treatment decisions and to assess the clinical usefulness of nutritional supplements and herbal medicine for improved well-being and quality of life in women with PCOS.

### Limitations

The inclusion of an English language excluded non-English language research and indexations. Herbal medicine has a high prevalence of use amongst non-English speaking cultures and this review was limited by its omission of non-English language research and databases.

Other limitations of this review include the poor quality of evidence, the small number of nutritional supplements and herbal medicine interventions, the absence of QoL and well-being outcomes and lack of long term investigations of safety. Key words did not include individual nutrient and herbal possibilities or complex herbal formulations and evidence may have been missed. In addition, methodological weaknesses lead to the exclusion of many studies and the authors did not seek further information from researchers of studies where the median rather than the mean was reported. Further research using rigorous RCT design is needed to inform findings of this systematic review.

A narrow inclusion criteria of only RCTs that examined the three diagnostic features of PCOS were specified for this review. Further literature reviews with inclusion criteria of other clinical outcomes such as fertility, anthropometric or psychological outcomes may strengthen the findings found here and better inform clinicians and women’s treatment decisions.

## Conclusion

There is no high quality evidence to support the effectiveness of nutritional supplements and herbal medicine for PCOS symptoms and evidence of safety and is lacking. However for two nutritional supplements, there was some low quality evidence that suggests women with PCOS may benefit from inositol and omega three fish oil supplements. Further research is needed.

## Additional file

Additional file 1: Table S1. studies excluded following full text review. (DOCX 30 kb)

## Abbreviations

BMI: Body mass index
CHM: Chinese herbal medicine
CI: Confidence interval
CM: Complementary medicine
CRP: C reactive protein
D3: Cholecalciferol
DHA: Docosahexaenoic Acid
EPA: Eicosapentaenoic acid
FAI: Free androgen index
FSH: Follicle stimulating hormone
HDL: High density lipoprotein
HOMA-IR: Homoeostatic model assessment of insulin resistance
IU: International units
LDL: Low density lipoprotein
LH: Luteinizing hormone
MD: Mean difference
NIH: National Institute of Health
OCP: Oral contraceptive pill
PCOS: Polycystic ovary syndrome
PRISMA: Preferred reporting items for systematic reviews and meta-analyses
PRL: Prolactin
QUICKI: Quantitative insulin sensitivity check index
RCT: Randomized control trial
RR: Relative risk
SHBG: Sex hormone binding globulin
sp.: Species
