# Supplementary material for: Nutritional supplements and herbal medicines for women with polycystic ovary syndrome; a systematic review and meta-analysis
Source: BMC Complement Altern Med. 2017 Nov 25;17:500. doi: 10.1186/s12906-017-2011-x (PMC5702141; doi:10.1186/s12906-017-2011-x)
Supplement: Additional file 1: Table S1. — studies excluded following full text review. (DOCX 30 kb) [file 12906_2017_2011_MOESM1_ESM.docx]

Supplementary Table 1 studies excluded following full text review

|  | **Authors** | **Title** | **Reference** | **Reason for exclusion** |
| --- | --- | --- | --- | --- |
| 1 | Ahmadi S, M Jamilian, M Karamali, M Tajabadi-Ebrahimi, P Jafari, M Taghizadeh, MR Memarzadeh and Z Asemi | Probiotic supplementation and the effects on weight loss, glycaemia and lipid profiles in women with polycystic ovary syndrome: a randomized, double-blind, placebo-controlled trial | Human Fertility 2017 1-8 | Primary outcomes were not investigated |
| 2 | Amooee S, ME Parsanezhad, MR Shirazi, S Alborzi and A Samsami | Metformin versus chromium picolinate in clomiphene citrate-resistant patients with PCOs: A double-blind randomized clinical trial | Iran Journal of Reproductive Medicine 2013,  11 (8) p. 611-618 | Trial not designed to investigate chromium supplements (chromium plus placebo verses chromium plus metformin) |
| 3 | Asemi Z, M Karamali and A Esmaillzadeh | Metabolic response to folate supplementation in overweight women with polycystic ovary syndrome: A randomized double-blind placebo-controlled clinical trial | Molecular and Nutritional Food Reserve 2014, 58  p. 1465-73 | Participants were not diagnosed with PCOS according to the Rotterdam or the NIH criteria |
| 4 | Asemi Z, F Foroozanfard, T Hashemi, F Bahmani, M Jamilian and A Esmaillzadeh | Calcium plus vitamin D supplementation affects glucose metabolism and lipid concentrations in overweight and obese vitamin D deficient women with polycystic ovary syndrome | Clinical Nutrition 2015, 34(4)  p. 586-592 | Primary outcome was not investigated |
| 5 | Bahmani F, M Karamali, H Shakeri and Z Asemi | The effects of folate supplementation on inflammatory factors and biomarkers of oxidative stress in overweight and obese women with polycystic ovary syndrome: a randomized, double-blind, placebo-controlled clinical trial | Clinical Endocrinology 2014, 81 p. 582-7 | Primary outcomes were not investigated. |
| 6 | Baillargeon, J-P, M J Iuorno, DJ Jakubowicz, T Apridonidze, N He and JE Nestler | Metformin therapy increases insulin-stimulated release of D-chiro-inositol-containing inositolphosphoglycan mediator in women with polycystic ovary syndrome | Journal of Clinical Endocrinology & Metabolism 2004, 89(1) p. 242-249 | Intervention did not align with inclusion criteria |
| 7a | Chen J, K Tominaga Y, Sato H, Anzai and R Matsuoka | Maitake mushroom (Grifola frondosa) extract induces ovulation in patients with polycystic ovary syndrome: a possible monotherapy and a combination therapy after failure with first-line clomiphene citrate. | The Journal of Alternative and Complementary Medicine 2010 16(12) p. 1295-1299 | Primary outcomes were not investigated |
| 8 | Ciotta L, M. Stracquadanio, I. Pagano, A. Carbonaro,  M. Palumbo and F. Gulino | Effects of Myo-Inositol supplementation on oocyte’s quality in PCOS patients: a double blind trial | European Review for Medical and Pharmacological Sciences 2011, 15 p.509-514 | Primary outcomes were not investigated. |
| 9 | Coskun A, T Arikan, M Kilinc, D.C. Arikan and H. Ç. Ekerbiçer | Plasma selenium levels in Turkish women with polycystic ovary syndrome | European Journal of Obstetrics & Gynecology & Reproductive Biology 2013, 168 (2) p.183-186 | Case controlled study |
| 10 | Foroozanfard F, M Jamilian†, F Bahmani, R Talaee, N Talaee,  T Hashemi, K Nasri, Z Asemi and A Esmaillzadeh | Calcium plus vitamin D supplementation influences biomarkers of inflammation and oxidative stress in overweight and vitamin D-deficient women with polycystic ovary syndrome: a randomized double-blind placebo-controlled clinical trial. | Clinical Endocrinology 2015, 83 p. 888–94 | Primary outcomes were not investigated. |
| 11a | Genzzani AD, C Lansoni, F Ricchieri and  VM Jasonni | Myo-inositol administration positively affects hyperinsulinaemia and hormonal parameters in overweight patients with polycystic ovary syndrome. | Gynecological Endocrinology 2008, 23 (4), p. 139-144 | Not a randomized controlled trial. |
| 12 | Haj-Husein H, S Tukan and F Alkazale | The effect of marjoram (Origanum majorana) tea on the hormonal profile of women with polycystic ovary syndrome: a randomised controlled pilot study | Journal of Human Nutrition and Dietetics 2016, 29  p. 105–111 | Participants aged 16 years were included in the trial. |
| 13a | Ismail AM, AH Hamed, S Saso, and HH Thabet | Adding L-carnitine to clomiphene resistant PCOS women improves the quality of ovulation and the pregnancy rate. A randomized clinical trial. | European Journal of Obstetrics and Gynaecology and Reproductive Biology. 2014, 180 p. 148-152 | Inclusion criteria didn’t specify minimum age of participants as 18 years. |
| 14 | Jalilian N, M Modarresi, M Rezaie, L Ghaderi and M Bozorgmanesh | Phytotherapeutic Management of Polycystic Ovary Syndrome: Role of Aerial Parts of Wood  Betony (Stachys lavandulifolia) | Phytotherapy Research  2013, 27  p. 1708–1713 | Diagnostic criteria of PCOS was not assessed according to Rotterdam or NIH criteria |
| 15 | Jamilian M, M Razavi, Z F Kashan, Y Ghandi, T Bagherian and Z Asemi | Metabolic response to selenium supplementation in women with polycystic ovary syndrome: a randomized, double-blind, placebo-controlled trial. | Clinical Endocrinology 2015, 82 p. 885-91 | Participant exclusion criteria didn’t exclude women with congenital adrenal hyperplasia |
| 16 | Jamilian H, M Jamilian, M Samimi, F Afshar Ebrahimi, M Rahimi, F Bahmani, S Aghababayan, M Kouhi, S Shahabbaspour and Z Asemi | Oral carnitine supplementation influences mental health parameters and biomarkers of oxidative stress in women with polycystic ovary syndrome: a randomized, double-blind, placebo-controlled trial | Gynecological Endocrinology 2017, 33 (6) p. 442-447 | Primary outcomes were not investigated |
| 17 | Kalgaonkar S, RU Almario, D Gurusinghe, EM Garamendi, W Buchan, K Kim and  SE Karakas | Differential effects of walnuts vs almonds on improving metabolic and endocrine parameters in PCOS | European Journal of Clinical Nutrition 2011, 65 p. 386-393 | Intervention was a food and not a type of ingestible supplement |
| 18 | Kamel, HH | Role of phyto-oestrogens in ovulation induction in women with polycystic ovarian syndrome | European Journal of Obstetrics & Gynecology and Reproductive Biology 2013, 168(1) p. 60-63 | Primary outcomes were not investigated. |
| 19 | Katcher, H I, AR Kunselman, R Dmitrovic, L M Demers, CL Gnatuk, PM Kris-Etherton and RS Legro | Comparison of hormonal and metabolic markers after a high-fat, Western meal versus a low-fat, high-fiber meal in women with polycystic ovary syndrome | Fertility & Sterility 2009, 91(4) p. 1175-1182. | Dietary interventions (western diet versus low fat, high fibre) |
| 20 | Kuek, SW, W J Gui | Efficacy of Chinese patent medicine Tian Gui Capsule in patients with polycystic ovary syndrome: a randomized controlled trial | Journal of Chinese Integrative Medicine 2011 9(9) p. 965-972. | Complex herbal formulation |
| 21 | Li,Y, H Kuang, W Shen, H Ma, Y Zhang, E Stener-Victorin, E Hung, Y Ng, J Liu, H Kuang, L Hou and X Wu | Letrozole, berberine, or their combination for anovulatory infertility in women with polycystic ovary syndrome: study design of a double-blind randomised controlled trial | BMJ Open 2013, **3**(11): e003934-e003934. | Investigated herbal chemical constituent Berberine |
| 22 | Maktabi M, M Chamani and Z Asemi | The Effects of Vitamin D Supplementation on Metabolic Status of Patients with Polycystic Ovary Syndrome: A Randomized, Double-Blind, Placebo-Controlled Trial. | Hormone and metabolic research 2017 49(7) p. 493 | Not truly randomized (matched pairs were randimized) |
| 23 | Mazloomi S, Sharifi F, Hajihosseini R,  Kalantari S, and Mazloomzadeh S | Association between hypoadiponectinemia and  low Serum Concentrations of Calcium and Vitamin D in women  with polycystic ovary syndrome | Gynecological Endocrinology 2012 27, (11) p.920-4 | Primary outcomes not investigated and participants aged less than 18 Not a randomized controlled trial |
| 24 | Moran, L. J., M. Noakes, P. M. Clifton, L. Tomlinson, C. Galletly and R. J. Norman | Dietary composition in restoring reproductive and metabolic physiology in overweight women with polycystic ovary syndrome | Journal of Clinical Endocrinology & Metabolism 2003 **88**(2) p. 812-819 | Dietary interventions |
| 25 | Moran L, S Zoungas, SA McNaughton, WJ Brown and HJ Teede | The contribution of diet, physical activity and sedentary behaviour to body mass index in women with and without polycystic ovary syndrome | Human Reproduction 2013 28(8) p.2276-83 | Dietary interventions |
| 26 | Papaleo E, V Unfer,J-P Baillargeon, F Fusi, F Occhi, and L De Santis | Myo-inositol may improve oocyte quality in intracytoplasmic sperm injection cycles. A prospective, controlled, randomized trial | Fertility and Sterility  2005, 91(5) p.1851-4 | Primary outcomes of review were not reported. |
| 27a | Phelan N, A O'Connor, T Kyaw Tun, N Correia, G Boran, HM Roche and J Gibney | Hormonal and metabolic effects of polyunsaturated fatty acids in young women with polycystic ovary syndrome: results from a cross-sectional analysis and a randomized, placebo-controlled, crossover trial | American Journal of Clinical Nutrition 2011, 93(3) p.652-662 | Primary outcomes not investigated |
| 28 | Rahimi-Ardabili, H, B Pourghassem Gargari and L Farzadi | Effects of vitamin D on cardiovascular disease risk factors in polycystic ovary syndrome women with vitamin D deficiency | Journal of Endocrinological Investigation 2013 **36**(1)p. 28-32 | Primary outcomes not investigated |
| 29 | Razavi M, M Jamilian, M Karamali, F Bahmani, E. Aghadavod and Z. Asemi | The Effects of Vitamin D-K-Calcium Co-Supplementation on Endocrine, Inflammation, and Oxidative Stress Biomarkers in Vitamin D-Deficient Women with Polycystic Ovary Syndrome: A Randomized, Double-  Blind, Placebo-Controlled Trial | Hormone Metabolism Research 2016, 47 (7) p. 446 | Not randomized control trial. |
| 30a | AY Rizk, MA Bedaiwy, and HG Al-Inany | N-acetyl-cysteine is a novel adjuvant to clomiphene citrate in clomiphene citrate–resistant patients with polycystic ovary syndrome | Fertility and Sterility  2005, 83 (2) p. 367 | Diagnostic criteria of PCOS was not assessed according to Rotterdam or NIH criteria (included women with polycystic ovaries only). |
| 31 | Samimi M, M Jamilian, FA Ebrahimi, M Rahimi, B Tajbakhsh and Z Asemi | Oral carnitine supplementation reduces body weight and insulin resistance in women with polycystic ovary syndrome: a randomized, double-blind, placebo-controlled trial. | Clinical Endocrinology 2016, 84 p. 854-857 | Participant exclusion criteria didn’t exclude women with congenital adrenal hyperplasia |
| 32a | Schachter M, A Raziel, D Strassburger, C Rotem, R Ron-El, S Friedler. | Prospective, randomized trial of metformin and vitamins for the reduction of plasma homocysteine  in insulin-resistant polycystic ovary syndrome | Fertility and Sterility 2007, 88 (1), p. 227 | Intervention administered as during an IVF cycle. Primary outcomes were not reported. |
| 33 | Shen, W, Y Zhang, W Li, J Cong, Y Zhou, EHY Ng and X Wu | Effects of tanshinone on hyperandrogenism and the quality of life in women with polycystic ovary syndrome: protocol of a double-blind, placebo-controlled, randomised trial | BMJ Open 2013, **3**(10): e003646-e0036462013 | Investigated a herbal chemical constituent |
| 34 | Shoaei T, M Heidari‑Beni, HG Tehrani, A Feizi, A Esmaillzadeh and G Askari | Effects of Probiotic Supplementation on Pancreatic β‑cell Function and C‑reactive Protein in Women with Polycystic Ovary Syndrome: A Randomized Double‑blind Placebo‑controlled  Clinical Trial | International Journal of Preventative Medicine 2015, 6 (24) p. 6:27. | Participants aged less than 18 years were included. |
| 35a | Thys-Jacobs S, D Donovan, A Papadopoulos, P Sarrel and JP Bilezikian | Vitamin D and calcium dysregulation in the polycystic ovarian syndrome | Steroids 1999, 64(6) p.1 430-435 | Not a randomized controlled trial |
| 36 | Unfer V, G Carlomagno, P Rizzo, E Raffone and S Roseff | Myo-inositol rather than D-chiro-inositol is able to improve oocyte quality in intracytoplasmic sperm injection cycles. A prospective, controlled, randomized trial | European Review for Medical & Pharmacological Sciences 2011, 15(4)  p. 452-457. | Primary outcomes were not investigated |
| 37 | Ushiroyama, T. I., A. Sakai, M. Hosotani, T. Suzuki, Y. Tsubokura, S. Ueki, M | Effects of unkei-to, an herbal medicine, on endocrine function and ovulation in women with high basal levels of luteinizing hormone secretion | Journal of Reproductive Medicine 2001, 46(5)  p. 451-4562001 | Complex herbal formulation was investigated. Participant inclusion criteria not Rotterdam or NIH. |
| 38 | Ushiroyama, T., T. Hosotani, Y. Yamashita, H. Yamashita and M. Ueki | Effects of Unkei-to on FSH, LH and estradiol in anovulatory young women with hyper- or hypo-functioning conditions | American Journal of Chinese Medicine 2003, 31(5) p. 763-771 | Complex herbal formulation. |
| 39 | Ushiroyama, T., T. Hosotani, K. Mori, Y. Yamashita, A. Ikeda and M. Ueki | "Effects of switching to wen-jing-tang (unkei-to) from preceding herbal preparations selected by eight-principle pattern identification on endocrinological status and ovulatory induction in women with polycystic ovary syndrome | American Journal of Chinese Medicine 34(2) p. 177-187 | Complex herbal formulation |
